# Daurisoline inhibits glycolysis of lung cancer by targeting the AKT-HK2 axis

**DOI:** 10.1080/15384047.2024.2442556

**Published:** 2024-12-19

**Authors:** Shi-Ming Tan, Lan Luo, Yi-Fu He, Wei Li, Xin-Xing Wan

**Affiliations:** a Department of Hematology, The Third Xiangya Hospital, Central South University, Changsha, China; b Department of Obstetrics and Gynecology, The Third Xiangya Hospital, Central South University, Changsha, China; c Department of Obstetrics and Gynecology, Xiangya Hospital, Central South University, Changsha, China; d Department of Radiology, The Third Xiangya Hospital, Central South University, Changsha, China; e Department of Endocrinology, The Third Xiangya Hospital, Central South University, Changsha, China

**Keywords:** Lung cancer, daurisoline, AKT, HK2

## Abstract

Lung cancer, one of the most prevalent tumors, remains a clinical challenge with a poor five-year survival rate. Daurisoline, a bis-benzylisoquinoline alkaloid derived from the traditional Chinese herb Menispermum dauricum, is known to suppress tumor growth effectively. However, its precise mechanism of action remains unclear. In this study, we demonstrate that Daurisoline targets glycolysis and reduces the protein level of HK2, thereby inhibiting lung cancer progression. Mechanistic investigations reveal that Daurisoline directly binds to AKT and antagonizes the AKT-GSK3β-c-Myc-HK2 signaling axis. Furthermore, in an animal model, we validate the in vivo anti-tumor effect of Daurisoline without any observable side effects. Overall, our findings suggest that Daurisoline holds potential as an anti-tumor agent through its targeting of glycolysis.

## Introduction

Lung cancer is one of the most prevalent and deadly malignancies, with the highest incidence and mortality rates among all malignant tumors worldwide.^1^ The treatment options for lung cancer are limited, and the survival rates are poor, especially for patients diagnosed at later stages.^2,3^ However, due to difficulties in early detection, many lung cancer cases are diagnosed at middle to late stages, leaving chemotherapy and radiotherapy as the primary treatment options, which result in poor five-year survival rates. Therefore, new drugs and treatment methods urgently need to be explored.^3–5^

Daurisoline is a bis-benzylisoquinoline alkaloid derivative from the rhizome of the traditional Chinese herb Menispermum dauricum.^6^ Bis-benzylisoquinoline alkaloids possess multiple pharmacological properties such as immunomodulation, anti-infection, antioxidant, anti-parasitic, and anti-inflammatory activities, but their mechanisms of action remain unclear.^7^ Previous studies have shown that bis-benzylisoquinoline alkaloids can induce antitumor effects in various cancers. The exact function of Daurisoline remains unclear, and its potential pharmacological effects need to be further confirmed. However, Daurisoline was also proven to strongly inhibit the growth of lung and hepatocellular carcinoma recently without the exact mechanism of its pharmacological effect,^8,9^ which should be of interest for further research.

The tumor microenvironment of lung cancer can accelerate progression of the disease, with lactate being a crucial metabolic product in this milieu.^10,11^ The high lactate concentration within the tumor microenvironment can curtail ubiquitination and degradation of HIF-1α by inhibiting prolyl hydroxylase (PHD) activity, leading to angiogenic sprouting.^12–14^ Lactate can drive primary tumor growth, chemoresistance, immune evasion, and metastasis through various signaling cascades such as HIF-1α, PKC, cAMP/PKA, and STAT3.^15–17^ Additionally, lactate can accelerate tumor progression in multiple tumors by activating GRP81.^18^ Therefore, it is essential to curtail lactate production in the tumor microenvironment. Most lactate in the body is produced via glycolysis, a rate-limited process by hexokinase (HK).^19^ Whether Daurisoline inhibits lung cancer through HK2 remains unclear, as the AKT-GSK3β pathway could regulate HK,^20^ and there are reports indicating that Daurisoline inhibits the activity of AKT.^20^ We postulate that Daurisoline can suppress lung cancer growth by regulating the AKT-GSK3β-HK axis that governs lactate production in the tumor microenvironment.

## Method and materials

### Cell culture and transfection

HCC827, H460 and H1299 cells were purchased from the American Type Culture Collection (ATCC) and cultured in DMEM (Life Technologies, Carlsbad, CA, USA) supplemented with 10% fetal bovine serum (FBS; Life Technologies) in a humidified incubator (37°C, 5% CO_2_) following standard protocols, and the Daurisoline (MCE, Shanghai, China), MK-2206 (MCE, Shanghai, China) was added to detect the proliferation and expression of the protein in HCC827, H460 and H1299 cells. The cells were detected for mycoplasma every two months routinely. When the cells were approximately 70% to 80% confluence, the c-Myc-T58A, Flag-HK2 plasmids (GeneChem, Shanghai, China) or AKT/GSK3β siRNAs (Ribio, Guangzhou, China) were transfected with Lipofectamine 3000 (Invitrogen, Carlsbad, CA, USA). After 48 hours, the cells were harvested for further experimentation.

### CCK assays

Lung cancer cells were seeded into 96-well plates (3×10^3^/well) and treated with different doses of Daurisoline. Cell viability was measured using the CCK8 reagent (Beyotime Institute of Biotechnology, Shanghai, China) following the standard protocol.

### Colony formation assay

The assay was performed as described previously.^21^ Cells were treated with DMSO or Daurisoline (2, 5, and 10 μmol/L) and incubated in six-well plates (500 cells/well) under normal conditions for 2 weeks. Visible colonies were fixed with 4% paraformaldehyde and stained with 0.5% crystal violet at 37°C. Colonies were counted using a light microscope.

### Soft agar assay

The soft agar assay was carried out as previously described.^22^ Lung cancer cells (8×10^3^/well) were suspended in 1 ml of 0.3% agar with Eagle’s medium (10% FBS, Life Technologies) and seed to six-well plates supplemented with 0.6% agar base. After culturing for 2 weeks, the images were captured by microscope.

### Glucose consumption and lactate assay

Measurement of glycolysis was performed as described previously.^23^ The differently treated lung cancer cells were seeded into 6-well plates and culture for 24 hours. The cell culture medium was collected to detect lactate production and glucose consumption in the Biochemical Laboratory of the Third Xiangya Hospital using an Automatic Biochemical Analyzer (7170A, HITACHI, Tokyo, Japan), with results normalized by protein concentration.

### In vitro AKT kinase analysis

The recombinant active AKT1, AKT2 and inactive GSK3β were purchased from Abcam (Palo Alto, CA, USA). The inactive GSK3β (1 μg) and active AKT1/2 (1 μg) were mixed with various doses of Daurisoline, followed by incubation with 10 μL of ATP mixture (25 mmol/L MgAc and 0.25 μmol/L ATP) for 15 min at 30°C and detected by immunoblotting.

### Immunohistochemical staining (IHC)

IHC staining was carried out as described previously.^24^ Tissues samples were fixed with 4% paraformaldehyde. The Department of Pathology, The Xiangya Hospital of Central South University carried out the following detection. The IHC (Solarbio, China) staining was performed according to the manufacturer’s protocol, and primary antibodies against HK2, p-AKT, and second antibodies were purchased from Proteintech, Wuhan, China.

### Isolation of mitochondrial fractions

The assay was performed as described previously.^21^ Briefly, the differently treated cells were harvested and washed once with ice-cold PBS and then resuspended in 3 volumes of isolation buffer (20 mmol/L HEPES, pH 7.4, 10 mmol/L KCl, 1.5 mmol/L MgCl_2_, 1 mmol/L sodium EDTA, 1 mmol/L dithiothreitol, 10 mmol/L phenylmethylsulfonyl fluoride, 10 mmol/L leupeptin and 10 mmol/L aprotinin) in 250 mmol/L sucrose. After chilling on ice, the cells were disrupted by 60 strokes of a glass homogenizer. The homogenate was centrifuged once at 2,000 rpm at 4°C for 10 min to remove unbroken cells and nuclei. The mitochondria-enriched fraction (supernatant) was then pelleted by centrifugation at 13,000 rpm for 30 min.

### Western blot assay

The experiment followed the procedure described in the published article of our laboratory.^25^ The total protein of cells was extracted and separated on a 12% sodium dodecyl sulfate -polyacrylamide gel electrophoresis (SDS-PAGE) gel and then transferred to nitrocellulose membranes with a transfer apparatus (Bio-Rad, Hercules, CA, USA). After blocking, primary antibodies, including HK2, HK1, cleaved-caspase 3, cytochrome C, Bax, VDAC1, p-AKT S473, AKT1/2, GSK3β, p-GSK3β, α-tubulin and β-actin (Proteintech, Wuhan, China) were treated overnight at 4°C. The secondary antibody was then applied, and protein detection was performed using enhanced chemiluminescence (ECL) (Advansta, Menlo Park, CA, USA).

### Co-immunoprecipitation (co-ip) assays

The cells were treated with IP lysis buffer (Life Technologies) according to the manufacturer’s instructions. Protein A/G-agarose beads (Santa Cruz Biotechnology, Japan) were pre-cleared and immunoprecipitated with 2 μg of survivin antibody at 4°C overnight. The next day, the cell lysates were incubated with the above beads at 4°C for 2 hours. The immunocomplexes and any co-immunoprecipitated proteins were then separated by SDS-PAGE and detected using the appropriate antibodies.

### Ex vivo pull-down assay

Daurisoline was conjugated to Sepharose 4B beads (Santa Cruz Biotechnology), according to the manufacturer’s instructions. Subsequently, HCC827 or H460 cell lysates were incubated with the control or Daurisoline-conjugated Sepharose 4B beads at 4°C for 12 h. A binding buffer was used to extract beads, which were then subjected to IB (immunoblot) assay.

### In vivo tumorigenicity assessment

The maintenance and experimentation of all mice were authorized by the Institutional Central South University (NO.476920210716, Changsha, China). Briefly, HCC827 (2 × 10^6^) cells were s.c.inoculated into the right flank of 6-week-old female thymus-free nude mice (Central South University) to create the xenograft mouse model. Tumor volume and mouse body weight were detected every two days. When the tumor volume reached 100 mm^3^, the mice with established tumors were randomly divided into two groups for the Daurisoline treatment (10 mg/kg every 4 days) or negative control. After Daurisoline treatment for 62 days, mice were euthanized, and the xenografts were removed, weighed, and then processed for immunohistochemical analysis.

### Blood assay

After Daurisoline treatment for 62 days, mice were euthanized, and blood was gathered from mice using EDTA-coated tubes. Red blood cell (RBC) and white blood cell (WBC) counts, along with alanine aminotransferase (ALT) and aspartate aminotransferase (AST) levels, were analyzed in the laboratory of Third Xiangya Hospital of Central South University, China.

### Statistical analysis

The results have been summarized and presented as mean ± standard deviation (SD). Statistical analysis was conducted using either a t-test or ANOVA, depending on the appropriateness of the statistical test for the specific data set. Statistical significance was determined at the levels of *p < .05, **p < .01, and ***p < .001.

## Results

### Daurisoline inhibits the growth of lung cancer

**Figure 1.**
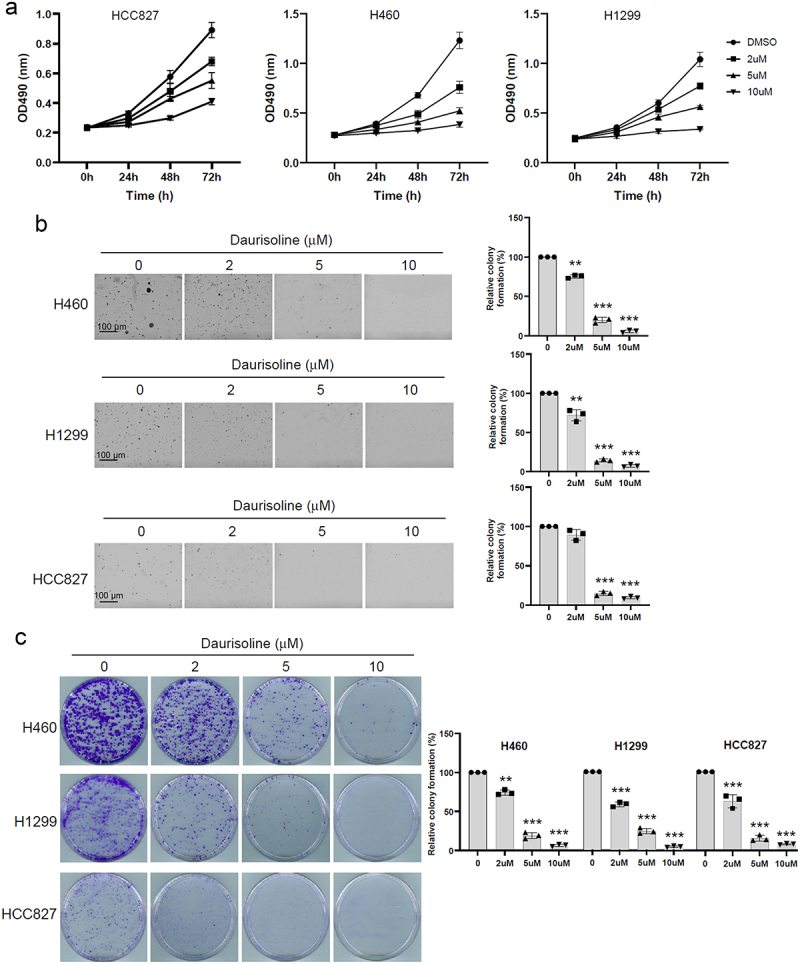
Daurisoline inhibits the proliferation of lung cancer cells.

We first detected whether Daurisoline can inhibit the growth of lung cancer. Different concentrations of Daurisoline were added to the mediums of HCC827, H460, and H1299. The CCK8 assay found that Daurisoline could decrease the proliferation of HCC827, H460 and H1299, and this function was dose and time-dependent (Figure 1a). As the same results, the soft agar clone and colony formation also proved that the proliferation of lung cancer cell lines was inhibited by Daurisoline (Figure 1b,c).

CCK8 (a), soft agar clone experiment (b), and colony formation assay (c) detected the proliferation of HCC827, H460 and H1299. *n* = 3, *p < .05, **p < .01, ***p < .001.

### Daurisoline decreased glucose consumption by inhibiting HK2

**Figure 2.**
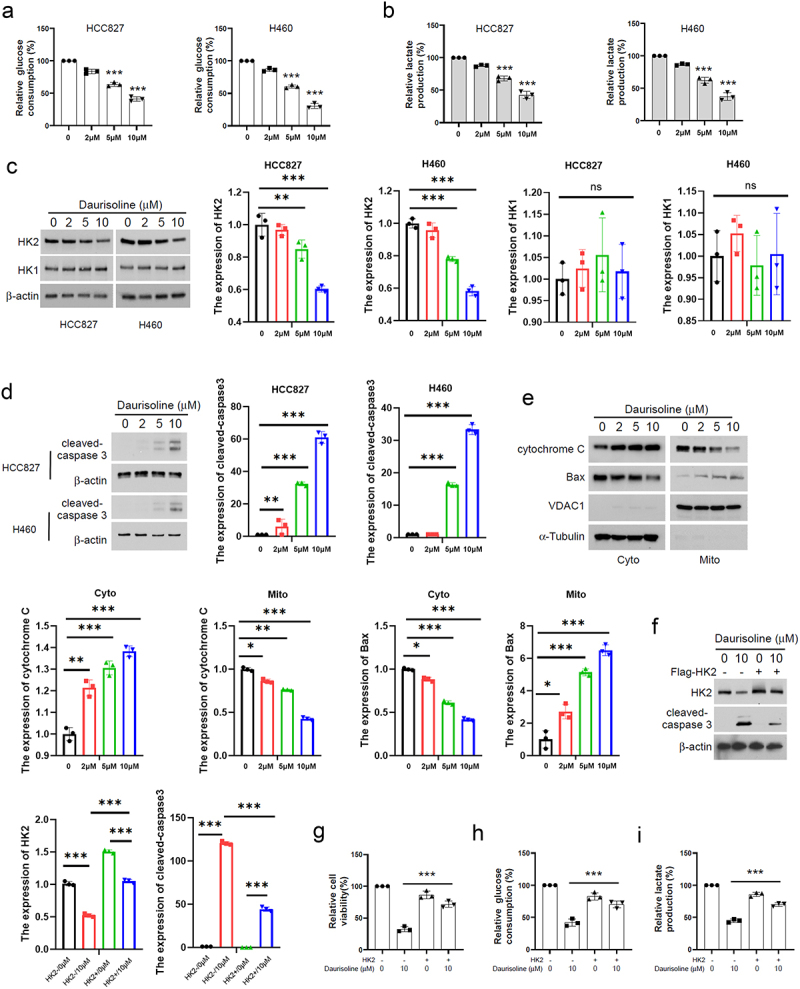
Daurisoline decreases glucose consumption. (a) Glucose consumption and lactate production (b) were detected using cell culture medium from HCC827 and H460. Western blot assay of the expression of (c) HK1 and HK2, (d) cleaved-caspase 3 and (e) cytochrome C and Bax in HCC827 and H460 treated with different concentrations of Daurisoline for 48 h. (f) Western blot assay detected the expression of cleaved-caspase 3 in the cells treated with 10 mmol/L Daurisoline with or without transfected with flag-HK2 plasmid for 48 h. CCK8(g), glucose consumption(h) and lactate production (i) were detected using cell culture medium from HCC827 treated with 10 mmol/L Daurisoline with or without transfected with flag-HK2 plasmid for 48 h. *n* = 3, *p < .05, **p < .01, ***p < .001.

The rapid growth of cancer cells can exaggerate the consumption of glucose and lactate production, which can indicate the growth rate of cancer cells. In this research, we detected the culture medium of HCC827 and H460 and proved that Daurisoline can decrease glucose consumption and lactate production in the lung cancer cells treated with Daurisoline (Figure 2a,b). As HK1 and HK2 were the key rate-limiting enzymes of glycolysis, we further detected the expression of HK1 and HK2 in the HCC827 cells. As Figure 2c,d showed, Daurisoline could decrease the expression of HK2 while the expression of HK1 was not affected by Daurisoline, and Daurisoline could elevate the expression of cleaved caspase 3 (Figure 2d). Subsequent Western blot assay revealed that cytochrome C was upregulated and Bax was downregulated in the cytoplasm of cancer cells treated with Daurisoline, but the contrary result occurred in the mitochondria of HCC827 cells (Figure 2e). We transfected Flag-HK2 plasmids in HCC827 and found increased HK2 can downregulate the cleaved-caspase 3 and reverse the viability, glucose consumption, and lactate production, which proved Daurisoline inhibited the proliferation of lung cancer dependent on HK2 (Figure 2f–i).

### Daurisoline inhibits HK2 through AKT-GSK3β pathway

**Figure 3.**
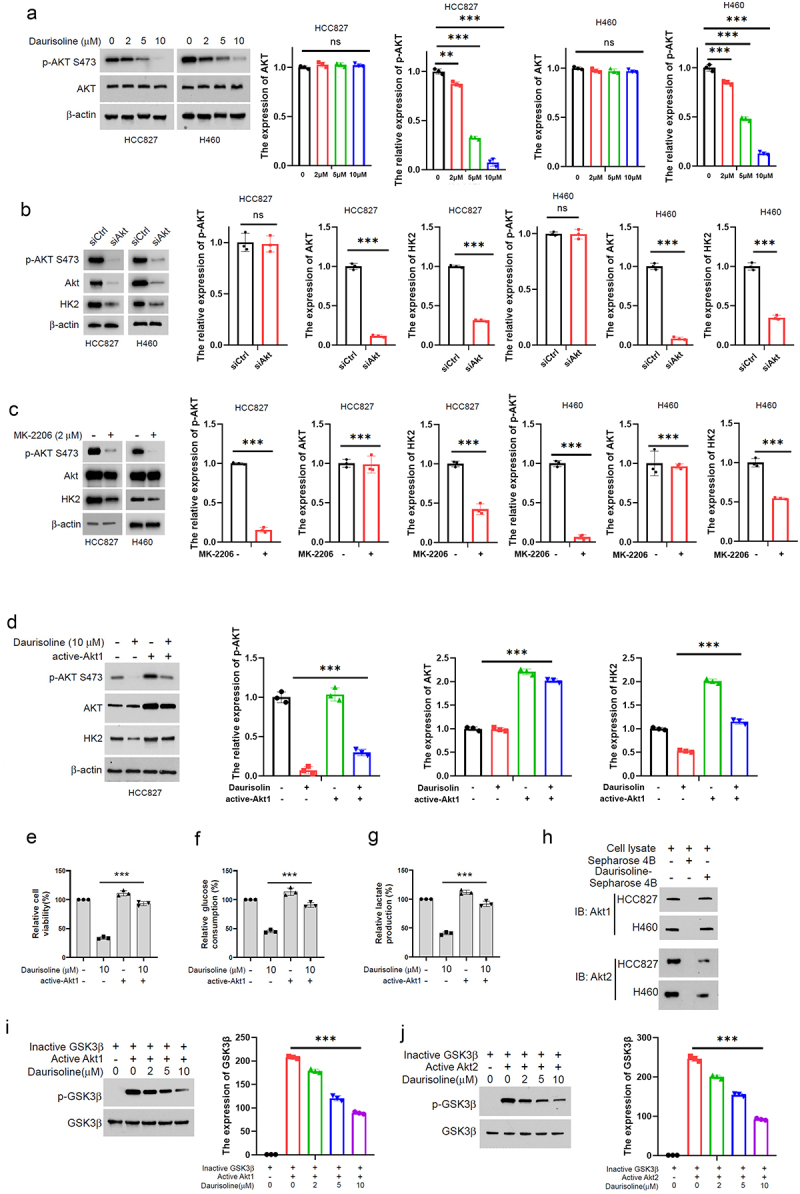
Daurisoline inhibits the HK2 by AKT. (a) AKT1 and phosphorylation -AKT1 was detected in the HCC827 and H460 treated with different concentrations of Daurisoline for 48 h. Western blot assay of the expression of HK2 in the HCC827 and H460 cells transfected with (b) Akt1 siRNA or (c) treated with MK-2206 after 48 h. (d) Western blot and (e) CCK8 assay of the expression of HK2 in the HCC827 and H460 cells treated with Daurisoline and active-Akt1 after 48 h. Assay of the expression of HK2. (f) glucose consumption and (g) lactate production assay in the HCC827 and H460 cells treated with Daurisoline and active-Akt1 (h) pull-down assay the combine between Akt1/Akt2 and Daurisoline. The immunity-blot assay of the expression phosphorylation – GSK3β treated with active (i) Akt1 or (j)Akt2. *n* = 3, *p < .05, **p < .01, ***p < .001.

The mechanism of Daurisoline inhibits the expression of HK2 was not clear. The AKT-GSK3β pathway has been shown to regulate the stability of HK2 in mitochondria. We found that the total expression of AKT was unchanged in HCC827 and H460 treated with Daurisoline, but the Ser-473-pho-AKT was inhibited by Daurisoline (Figure 3a). Similarly, the HCC827 and H460 treated with MK-2206 AND silenced by AKT siRNA could both downregulate the expression of HK2 (Figure 3b,c), and the activation of AKT1 can increase the expression of HK2, recover the viability, glucose consumption and lactate production in the HCC827 cell (Figure 3d-g). Further experiments revealed that Daurisoline can be combined with Ak1 and Akt2 (Figure 3h), which explained that the activity of GSK3 decreased when treated with Daurisoline and Akt1/Akt2 (Figure 3i,j).

### Daurisoline downregulated c-myc by enhancing ubiquitination but increased the phosphorylation of c-myc at T58

**Figure 4.**
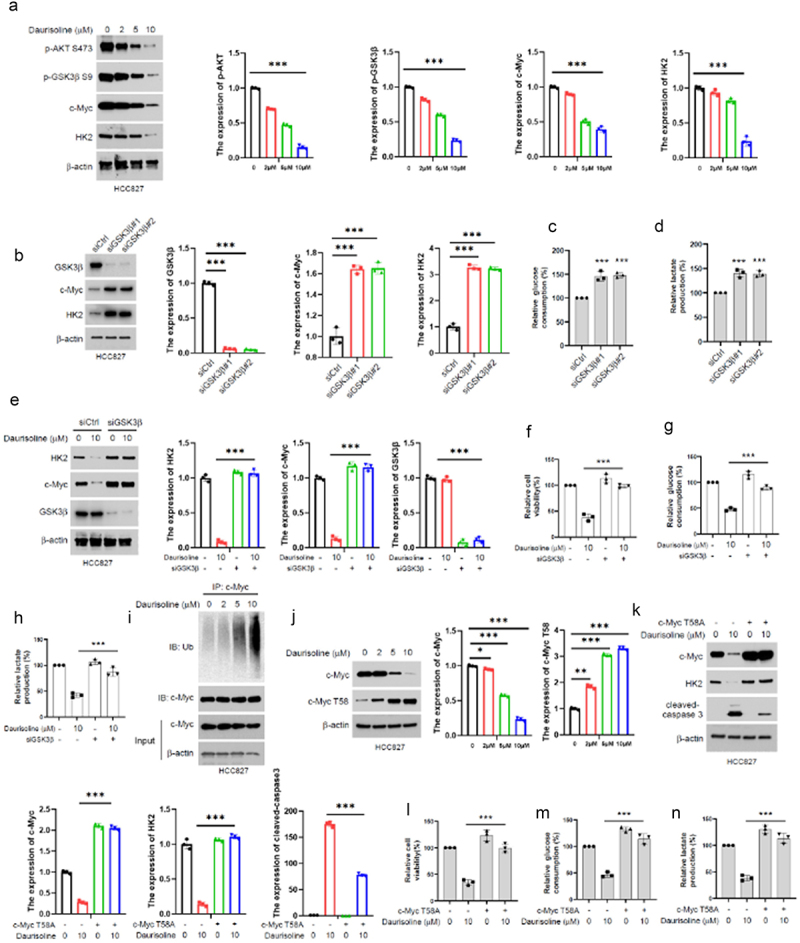
Daurisoline increases the ubiquitination of c-myc. (a) Western blot assay of the expression of C-myc in HCC827 treated with daurisoline after 48 h. (b) Western blot assay of the expression of C-myc/HK2 after transfected siGsk3β for 48 h, the (c) glucose consumption and (d) lactate production assay in the HCC827 transfected with GSK3β siRNA for 48 h. (e) Western blot assay of the expression of C-myc/HK2, and (f) CCK8 assay of the cell viability after 48 h treated with daurisoline and tranfected with GSK3β siRNA, (g) glucose consumption and (h) lactate production assay in the HCC827 transfected with GSK3β siRNA for 48 h. (i) IP assay the ubiquitination of c-myc in HCC827 treated with daurisoline. (j) Western blot assay of c-myc and cMyc T58 expression in HCC827 treated with daurisoline. (k) Western blot assay of the expression cleaved-caspase 3 and (l) CCK8 assay the cell viability in HCC827 transfected with c-myc T58A plasmid after 48 h. (m) glucose consumption and (n) lactate production assay in the HCC827 transfected with c-myc T58A plasmids after 48 h. *n* = 3, *p < .05, **p < .01, ***p < .001.

Phosphorylation of GSK3β at Ser9 has been shown to regulate the expression and activity of c-Myc. Therefore, we aimed to investigate whether Daurisoline inhibits c-Myc through the AKT-GSK3β pathway. Figure 4a shows that Daurisoline inhibits p-AKT S473, p-GSK3β S9, and c-Myc in a dose-dependent manner. Decreased expression of GSK3β enhances the expression of c-Myc, increases glucose consumption and lactate production (Figure 4b-d), and reverses the tumor-suppressive effects of Daurisoline, such as inhibition of cell viability, glucose consumption, and lactate production (Figure 4e-h). Mechanistically, we demonstrated that Daurisoline decreases the expression of c-Myc by enhancing its ubiquitination, while increasing the phosphorylation of c-Myc at T58 (Figure 4i,j). Further studies confirmed that transfection of the c-Myc T58A mutant plasmid abrogated the effects of Daurisoline (Figure 4k-n).

### Daurisoline attenuate tumor growth in nude mice without noticeable side effect

**Figure 5.**
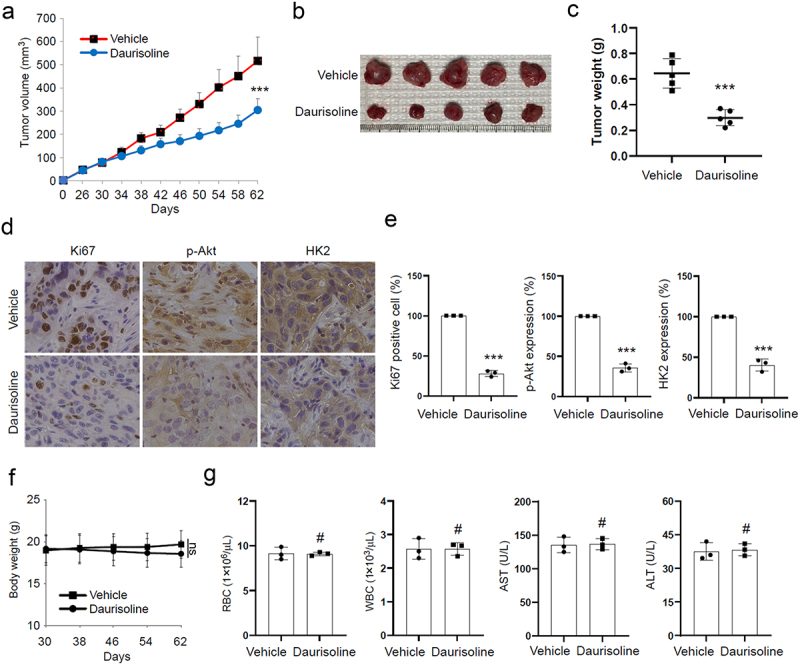
Daurisoline inhibits lung cancer in mice, *n* = 5. (a) The tumor volume in nude mice treated with Daurisoline, *n* = 5. (b&c) the tumor weight in nude mice treated with Daurisoline. (d&e) immunohistochemistry detects the Ki67, p-Akt, and HK2 in the mice. (f&g) the body weight and blood biochemical index assay of mice mode. *n* = 3, *p < .05, **p < .01, ***p < .001.

As a potential anti-tumor compound, we continued to research the effect of Daurisoline in the animal model. After we seeded HCC827 and HCC827 combined with Daurisoline to the nude mice, the tumor volume was observed and measured every four days after 26 days, and the results of Figure 5a showed tumors in the Daurisoline-treated group were smaller compared to the control group. The tumor tissues of sacrificed mice were collected for further research. Both tumor volume and weight were significantly reduced in the Daurisoline-treated group compared to the vehicle control (Figure 5b,c). Moreover, the IHC experiment found reduced expression of Ki67, p-Akt and HK2 in the tumor tissue of HCC827 combined with Daurisoline (Figure 5d,e). Notably, no significant side effects were observed in terms of body weight or blood tests.

## Discussions

Lung cancer is one of the most prevalent tumors, characterized by its high degree of malignancy, rapid growth, and tendency toward metastasis. The treatment of this disease in the clinic is complex, and the five-year survival rate is dismally low in patients with lung cancers, which significantly impacting their quality of life.^26^ Due to the limited efficacy of traditional treatments such as surgery, chemotherapy, and radiotherapy for lung cancer, many innovative therapies such as gene-targeted therapy, immunotherapy, and Chinese herbal extracts treatments, have shown promising results in treating lung cancer.^27,28^

Although various researchers declared bis-benzylisoquinoline alkaloid could potently inhibit the growth of different types of cancer, only a few researches were focused on the relationship between Daurisoline and lung cancer.^29^ Daurisoline extracted from the rhizome of the traditional Chinese herb Menispermum dauricum is a bis-benzylisoquinoline alkaloid.^30^ We hypothesize that Daurisoline can hinder the progression of lung cancer. To identify this hypothesis, we employed three lung cancer cell lines treated with varying concentrations of Daurisoline, demonstrating that Daurisoline can suppress the proliferation of lung cancer cells (Figure 1).

The tumor microenvironment plays a crucial role in maintaining the proliferation and invasive capacity of tumors.^31^ Lactate stands as a prominent metabolite resulting from glycolysis.^32^ Tumor cells exhibit rapid growth and necessitate a substantial energy supply, leading them to produce vast quantities of lactate via the Warburg effect, as opposed to typical aerobic glucose consumption.^33–35^ High lactic acid levels in the tumor microenvironment can protect cells from immune cell attacks, further promoting tumor growth and metastasis.^36^ Altering glucose metabolism in lung cancer is important for treating lung cancer. HK is the key rate-limiting enzyme of glycolysis, and it phosphorylates glucose to initiate its conversion into energy.^37^ HK1 and HK2 are the primary subtypes of HK.^38^ The results presented in Figure 2 demonstrate that Daurisoline can reduce the expression of HK2, but not HK1, in lung cancer cell lines. This reduction is accompanied by decreased glucose consumption and lactate production. Additionally, Daurisoline can increase the expression of cleaved-caspase 3. It has been established that HK2 binds to the outer mitochondrial membrane voltage-dependent anion channel (VDAC) in mitochondria, stabilizing the cell and enhancing ATP production, thereby supporting tumor cell growth. Conversely, reducing HK2 expression promotes cytochrome C release into the cytoplasm, ultimately leading to apoptosis in tumor cells.^39,40^ The results in Figure 2e imply that Daurisoline increases the apoptosis of lung cancer through HK2-mediated cytochrome C, which was further validated by the observation that overexpressing HK2 reversed the effect of Daurisoline in HCC827 cells.

These findings provide insights into the potential therapeutic role of Daurisoline in lung cancer treatment through its modulation of glucose metabolism and mitochondrial function. However, further studies are required to fully understand the exact mechanism behind Daurisoline’s regulation of HK2 expression. Additionally, assessing the safety and efficacy of Daurisoline as a potential anti-tumor agent in pre-clinical models and clinical trials is essential before any conclusions can be drawn regarding its use as a potential therapy for lung cancer. HK2 has been proven to be active by the PI3K-AKT pathway in many reports.^41^ The plant-derived alkaloids could mediate various types of death, such as apoptosis, ferroptosis, and autophagy, and regulate the cancer progression depending on the tumor microenvironment.^42^ The tumor microenvironment can be modulated by the PI3K pathway,^43^ suggesting a potential relationship between the PI3K pathway and HK2. Recent studies have demonstrated that glucose uptake and lactate production decrease when transfected with PI3K siRNA, indicating that the PI3K-AKT pathway may be a key regulator of HK2. These founding reminders that Daurisoline could also inhibit the HK2 through the AKT pathway are proved in our result in Figure 3a-g.

AKT is a central molecular player in many metabolic pathways, and alterations in its activation status can cause various diseases.^44,45^ We further demonstrated that Daurisoline can directly bind to AKT1 and AKT2, thereby inhibiting their phosphorylation of GSK3β (Figure 3h-j). Interestingly, although subsequent research found that Daurisoline can decrease the phosphorylation of GSK3β at S9, silencing GSK3β expression using siRNA can increase glucose and lactate levels, providing evidence for the dependence of Daurisoline’s function on GSK3β phosphorylation at S9. C-Myc is a downstream protein of the AKT-GSK3β pathway, which can regulate the growth, proliferation, survival and differentiation of tumor cells.^46,47^ In addition, c-Myc can regulate the tumor microenvironment by modulating the secretion of cytokines through innate and adaptive immune systems. The inactivation of c-Myc could induce sustained tumor regression.^48^ It is necessary to explore whether Daurisoline could regulate the activity of c-Myc. Then we found that Daurisoline has restrained expression of c-Myc through ubiquitination-mediated degradation (Figure 4i-j), but the phosphorylation of c-Myc T58 was increased, and transfected T58A mutant c-Myc could reserve the inhibit function of Daurisoline (Figure 4k). Animal experiments are essential for pharmacological mechanisms, and after we completed cytological experiments, we conducted further in vivo studies on Daurisoline and demonstrated that Daurisoline can inhibit lung cancer in nude mice with no significant toxic side effects (Figure 5).

## Conclusion

In general, our results confirmed that the AKT-GSK3β-c-Myc-HK2 was the critical pathway regulated by the dose-dependent Daurisoline in lung cancer cells, and we proved Daurisoline was the potential therapeutic oncology drug.

## Data Availability

The data used to support the findings of this study are available from the corresponding author upon request.
